# Sanguinarine chloride induces ferroptosis by regulating ROS/BACH1/HMOX1 signaling pathway in prostate cancer

**DOI:** 10.1186/s13020-024-00881-6

**Published:** 2024-01-09

**Authors:** Shanhui Liu, Yan Tao, Shan Wu, Jiawei Lin, Shengjun Fu, Jianzhong Lu, Jing Zhang, Beitang Fu, Erdong Zhang, Jing Xu, Jiaxuan Wang, Lanlan Li, Lei Zhang, Zhiping Wang

**Affiliations:** 1 Institute of Urology, Clinical Research Center for Urology in Gansu Province, Key Laboratory of Urological Disease in Gansu Province, Lanzhou University Second Hospital, No. 82 Cuiyingmen, Lanzhou, 730030 Gansu China; 2 Gansu Provincial Center for Disease Control and Prevention, Lanzhou, 730000 Gansu China; 3 Cyrus Tang Hematology Center, Collaborative Innovation Center of Hematology, National Clinical Research Center for Hematologic Diseases, Soochow University, Suzhou, 215123 Jiangsu China; 4 The Fifth Affiliated Hospital of Xinjiang Medical University, Ürümqi, 830000 China; 5 Key Laboratory of Optimal Utilization of Natural Medicinal Resources, Guizhou Medical University, Guiyang, 550025 Guizhou China; 6 The Second Clinical Medical College of Lanzhou University, Lanzhou University, Lanzhou, 730000 Gansu China

**Keywords:** Ferroptosis, Sanguinarine chloride, Prostate cancer, HMOX1, BACH1

## Abstract

**Background:**

Sanguinarine chloride (S.C) is a benzophenanthrine alkaloid derived from the root of sanguinaria canadensis and other poppy-fumaria species. Studies have reported that S.C exhibits antioxidant, anti-inflammatory, proapoptotic, and growth inhibitory effects, which contribute to its anti-cancer properties. Recent studies suggested that the antitumor effect of S.C through inducing ferroptosis in some cancers. Nevertheless, the precise mechanism underlying the regulation of ferroptosis by S.C remains poorly understood.

**Methods:**

A small molecule library was constructed based on FDA and CFDA approved small molecular drugs. CCK-8 assay was applied to evaluate the effects of the small molecule compound on tumor cell viability. Prostate cancer cells were treated with S.C and then the cell viability and migration ability were assessed using CCK8, colony formation and wound healing assay. Reactive oxygen species (ROS) and iron accumulation were quantified through flow cytometry analysis. The levels of malondialdehyde (MDA) and total glutathione (GSH) were measured using commercially available kits. RNA-seq analysis was performed to identify differentially expressed genes (DEGs) among the treatment groups. Western blotting and qPCR were utilized to investigate the expression of relevant proteins and genes. In vivo experiments employed a xenograft mice model to evaluate the anti-cancer efficacy of S.C.

**Results:**

Our study demonstrated that S.C effectively inhibited the viability of various prostate cancer cells. Notably, S.C exhibited the ability to enhance the cytotoxicity of docetaxel in DU145 cells. We found that S.C-induced cell death partially relied on the induction of ferroptosis, which was mediated through up-regulation of HMOX1 protein. Additionally, our investigation revealed that S.C treatment decreased the stability of BACH1 protein, which contributed to HMOX1expression. We further identified that S.C-induced ROS caused BACH1 instability by suppressing USP47expression. Moreover, In DU145 xenograft model, we found S.C significantly inhibited prostate cancer growth, highlighting its potential as a therapeutic strategy. Collectively, these findings provide evidence that S.C could induce regulated cell death (RCD) in prostate cancer cells and effectively inhibit tumor growth via triggering ferroptosis. This study provides evidence that S.C effectively suppresses tumor progression and induces ferroptosis in prostate cancer cells by targeting ROS/USP47/BACH1/HMOX1 axis.

**Conclusion:**

This study provides evidence that S.C effectively suppresses tumor progression and induces ferroptosis in prostate cancer cells by targeting the ROS/USP47/BACH1/HMOX1 axis. These findings offer novel insights into the underlying mechanism by which S.C inhibits the progression of prostate cancer. Furthermore, leveraging the potential of S.C in targeting ferroptosis may present a new therapeutic opportunity for prostate cancer. This study found that S.C induces ferroptosis by targeting the ROS/USP47/BACH1/HMOX1 axis in prostate cancer cells.

**Graphical Abstract:**

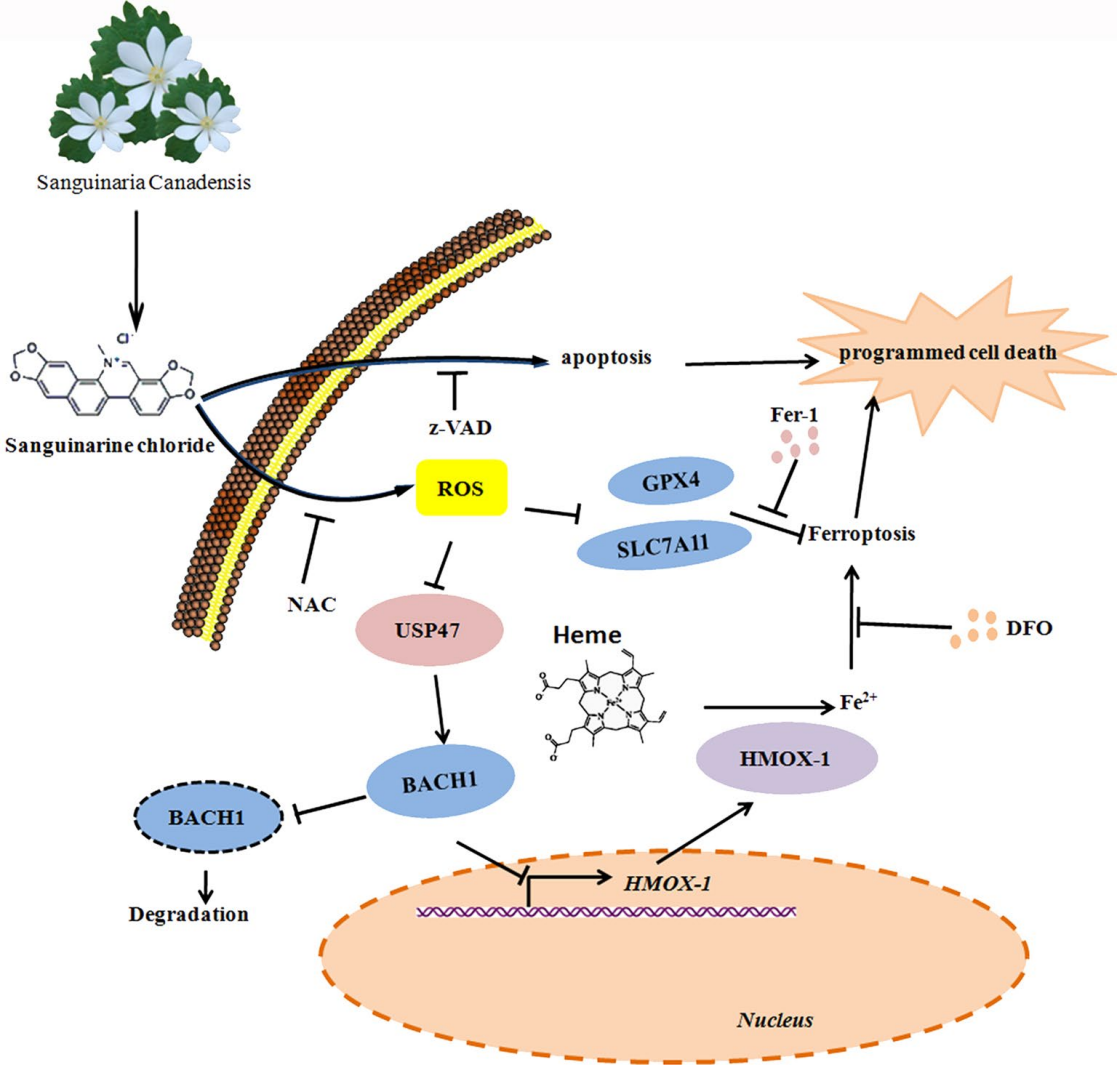

**Supplementary Information:**

The online version contains supplementary material available at 10.1186/s13020-024-00881-6.

## Introduction

Prostate cancer (PCa) is the most common cancer and remains a leading cause of cancer-related deaths among men worldwide [1–3]. The treatment strategies for localized PCa mainly depend on targeting AR (androgen receptor) signaling as it is predominantly activated during PCa progression [4]. Several inhibitors targeting this pathway (e.g. enzalutamide/abiraterone/apalutamide/bicalutamide. etc.) have been successfully used to constrain tumor progression. However, the efficacy of these treatments is typically limited, which leads to the emergence of drug resistance and tumor recurrence. Most PCa patients ultimately develop to the form of castration-resistant prostate cancer (CRPC), which only has a short median survival time of about 14 months [5]. Hence, effective therapeutic options to overcome the drug resistance are urgently desired.

Ferroptosis is a novel type of regulated cell death [6]. Distinct from apoptosis, necroptosis, pyroptosis, and autophagy, it is characterized by the cellular accumulation of iron and toxic lipid peroxides [7]. Ferroptosis can be triggered by abnormal expression of transporters, such as SLC7A11 inhibition or transferrin and lactotransferrin activation, as well as antioxidant enzymes like GPX4 modulation. Small molecules, such as erastin, JKE-1674 and GPX4-IN-3, as well as various stress conditions like high temperature, low temperature, hypoxia, and radiation, could also induce ferroptosis [8]. Extensive studies have demonstrated that ferroptosis induction is a promising therapeutic strategy to inhibit cancer progression and overcome drug resistance in cancer cells [9, 10]. In the context of prostate cancer, the androgen-repressed gene DECR1 has been implicated in the development of castration-resistant prostate cancer (CRPC) and resistance to androgen receptor (AR)-targeted treatments through regulating polyunsaturated fatty acids (PUFAs) oxidation. Targeting DECR1 disrupts PUFA oxidation and induces ferroptosis in both PCa and CRPC [11]. This suggests that inducing ferroptosis could overcome resistance to anti-androgens therapy resistance in PCa. Notably, either inducing ferroptosis alone or synergizing with anti-androgen agents have shown significant efficacy in halting cell progression in PCa and CRPC [12].

Sanguinarine chloride (S.C) is a natural benzophenanthridine alkaloid extracted from various sources, including the roots of Sanguinaria canadensis, seeds of Argemone Mexicana, and the leaves and fruits of Macleaya cordata [13]. Numerous studies have reported its pharmacological activity in cancer therapy [13–15]. Recently investigations have highlighted that the anti-cancer activity of sanguinarine mainly depends on inducing the generation of reactive oxygen species and suppressing the JAK/STAT pathway [14, 16–18]. Additionally, sanguinarine displayed anti-metastatic properties and the ability to reverse epithelial-to-mesenchymal transition in estrogen receptor-positive (ER +) breast cancer [19]. Notably, sanguinarine has displayed effectiveness against multidrug resistance in human cervical and ovarian cancer cells [13, 20].Evidence suggests that sanguinarine may contribute to ferroptosis by reducing intracellular glutathione content in cisplatin-resistant ovarian cancer cells. As the Xc − /GSH/GPX4 axis serves as an important antioxidant system in ferroptosis by catalyzing the reduction of lipid peroxides, sanguinarine might induce ferroptosis in cancer cells through the reduction of intracellular glutathione levels. In fact, recent study demonstrated that sanguinarine could induce ferroptosis in human cervival cancer cells in an H_2_O_2_-dependent manner [13]. Despite these discoveries, the detailed understanding of the pharmacological actions of S.C on prostate cancer and castration-resistant prostate cancer, as well as the intricate molecular signaling mechanisms involved in ferroptosis, remains limited.

In this study, we aim to explore the role of S.C in prostate cancer (PCa) therapy. This study demonstrated that S.C effectively decreased the viability, clonogenicity, and tumorigenicity of PCa cells both in vitro and in vivo. In addition to inducing intrinsic apoptosis, S.C triggered ferroptosis in PCa cells, as evidenced by intracellular iron overload, MDA overexpression, and ROS accumulation. Mechanistically, S.C induced intracellular iron overload by up-regulating HMOX-1, a critical enzyme in heme breakdown, leading to heme degradation and the release of labile Fe^2+,^ a pivotal initiator of ferroptosis [21, 22]. Additionally, our study uncovered the significant role of ROS in S.C-induced HMOX-1 expression. In this process, ROS may reduce the stability of BACH1, which binds to the HMOX1 promoter region, consequently suppressing its transcription in a USP47-dependent manner. Overall, our findings shed light on the pharmacological effects of S.C in PCa therapy, especially in the context of castration-resistant prostate cancer (CRPC), and present a promising new approach for treating PCa.

## Materials and methods

### Materials

The prostate cancer cell lines LNCaP, VCaP, 22RV1, PC3 and DU145 were obtained from the Chinese Academy of Sciences Cell Bank of Type Culture Collection. Enzalutamide resistant cell including LNCaP-enz and 22RV1-enz were kept in our lab. Sanguinarine chloride (T0129), Enzalutamide (T6002), Docetaxel (T1034), z-VAD-fmk (T7020), Ferrostatin-1 (T6500) Deferoxamine Mesylate (T1637) and all components in ourcompound library were purchased from Targetmol. CCK8 reagent (CYT001) was purchased from Yoche. N-Acetyl-L-cysteine (ST2524), Lipid Peroxidation MDA Assay Kit (S0131S), Total Glutathione Peroxidase Assay Kit with NADPH (S0058) and Annexin V-FITC Apoptosis Detection Kit (C1062L) were purchased from Beyotime. Mito-FerroGreen kit (M489) was purchased from Dojindo. Cycloheximide (HY-12320) was purchased from MedChem Express. The primary antibodies used for IHC and Western blotting were following: GAPDH (ab128915), caspase-9 (ab32539) and BAX (ab32503) were purchased from abcam. BIM (A19702), HMOX1 (A11102), BACH1 (A5393), SLC7A11/xCT (A2413), GPX4 (A1933), KEAP1 (A21724), PERK (A18196), USP47 (A15461), Histone H3 (A2348) and ki67 (A21861) were purchased from Abclonal. HIF1α (AF1009) was purchased from affinity. BCL2 (2872), STAT3 (12640S), PARP (9532), cleaved-caspase3 (9664S) and BAK (12,105) were purchased from Cell Signaling Technology. BCL-XL (66,020-1-Ig), caspase 8 (66,093-1-Ig), caspase 9 (66,169-1-Ig), MCL1 (66,026–1-Ig) and NRF2 (16,396-1-AP) were purchased from proteintech.

### Cell culture

LNCaP, VCaP, 22RV1, PC3, DU145,LNCaP-enz and 22RV1-enz cells were cultured in RPMI1640 medium or Dulbecco’s modified Eagle’s medium supplemented with 10% fetal bovine serum, 100 U/ml penicillin and 0.1 mg/ml streptomycin at 37 ℃ with 5% CO_2_.

### Plasmid construction and transfection

All overexpression plasmid (pcDNA3.1-*HMOX1* and pcDNA3.1-*BACH1*) was provided by Tsingke Biotechnology. *HMOX1* knockdown vectors (pLKO.1-*HMOX1-a/b/c*) were synthesized by Synbio-Technologies. The target sequences of sh-*HMOX1-a/b/c* were as following: sh-*HMOX1-a*, 5ʹ—cctccctgtaccacatctatg—3ʹ; sh-*HMOX1-b*, 5ʹ—gctcaacatccagctctttga—3ʹ; sh-*HMOX1-c*, 5ʹ—acagttgctgtagggctttat—3ʹ. Lentivirus was produced by transfection of PLKO.1 vectors together with the packaging plasmid psPAX.2 and the envelope plasmid pMD2.G into HEK293T cells. Virus-containing supernatant was collected at 48 h after transfection. DU145 and PC3 cells were infected with lentiviral supernatants in the presence of polybrene for 12 h. These infected cells were further selected for two days with puromycin (8 μg/ml).

### Wound-healing assay

PC3 and DU145 cells were pre-treated with 0, 0.5, 1.0 µM S.C for 24 h. Cells were then plated into 6-well plate at a density of 5 × 10^5^ cells/well and were cultured till to 90% confluence. A wound in each well was created with pipette tip. Cells were then further cultured with serum-free medium for 0 h and 24 h. Wound-healing process was photographed at each time point.

### Cell viability assay

Cells were plated into 96-well plates and treated with indicated compounds for 24 h or 48 h. CCK8 reagent was added into wells and incubated for 2–4 h. Subsequently, the absorbance at 450 nm was measured by a microplate reader.

### Clonogenic assays

The clonogenic assay was performed to assess the clonogenicity capabilities of the indicated cells, following a previously described protocol [23]. Briefly, cells were treated with varying concentrations of S.C (0, 0.1, 0.5, and 1 μM) and allowed to grow for ten days, with regular changes of fresh medium every 3–4 days. Following removal of the media, the colonies were washed with ice-cold PBS, fixed with 4% paraformaldehyde, stained with crystal violet solution for 15 min at room temperature, and rinsed with distilled water to remove excess dye. The resulting colonies were then counted for each sample.

### Hoechst 33,258 staining

PC3 and DU145 cells were seeded into 24-well plates and treated with S.C at concentrations of 0.5 μM and 1.0 μM for either 24 h or 48 h. Subsequently, the cells were fixed with 75% ethanol, stained with Hoechst 33,258 solution, and visualized using a fluorescence microscope.

### mRNA expression analysis by real-time PCR

mRNA expression levels were analyzed following a previously described protocol [23]. Briefly, total RNAs were extracted from the stimulated prostate cancer cell lines using TRIzol reagent (Takara Biotechnology Co., Dalian, China). Subsequently, the extracted RNA was converted into cDNA using the PrimeScript RT reagent kit (Takara, Dalian, China). The expression levels of the target genes were quantified using the SYBR Premix Ex Taq kit (Takara, Dalian, China) and the Bio-Rad CFX-96 thermal cycler(Bio-Rad, Hercules, CA). Normalization of the target gene mRNA expression levels was achieved by co-amplification of *β-actin*. Primers sequences used for real-time PCR were: *HMOX1* (forward:5ʹ—gctatgtgaagcggctccac—3ʹ; reverse: 5ʹ—cagggctttctgggcaatc—3ʹ);*β-actin* (forward: 5ʹ—gcacagagcctcgcctt—3ʹ; reverse:5ʹ—gttgtcgacgacgagcg-3ʹ).

### Western blotting assay

Prostate cancer cell lines were treated in vitro as indicated and lysed in the cell lysis buffer (catalog no. P0013B; Beyotime Biotechnology, China) presented with protease /phosphatase inhibitor cocktail (catalog no. 5872; Cell Signaling Technology, Danvers, MA). The lysate were quantified with the bicinchoninic acid (BCA) kit (Beyotime Biotechnology, China). Then, the supernatants were subjected to 10% SDS-PAGE gel and then transferred to PVDF membranes (0.45 μm, Amersham, cat no. 10600023) for detection of NRF2,STAT3, BACH1, PERK, KEAP1, HIF-1a, USP47, H3, HMOX1, SLC7A11, GPX4, PARP, pro-caspase3, caspase3, pro-caspase9, caspase9, caspase8, BCL2, BAK, BIM, BAX and GAPDH proteins.

### Malondialdehyde (MDA) and total glutathione peroxidase assay

PC3 and DU145 cells were plated in 100 mm dishes and treated with DMSO or S.C for 48 h. Then, the contents of MDA and total glutathione peroxidase level were quantified using the Lipid Peroxidation MDA Assay Kit (S0131S, Beyotime, China) and Total Glutathione Peroxidase Assay Kit (S0058, Beyotime, China) in accordance to the manufacturer’s instructions.

### Annexin V-FITC–propium iodide assay

VCaP, PC3 and DU145 cells were plated in 60 mm dishes and treated with DMSO or S.C (1.0 μM)for 48 h. Then, cells were used to determine number of apoptotic cells with the Annexin V-FITC apoptosis kit according to the manufacture’s instruction (C1062L, Beyotime, China). Cells were subjected to flow cytometry (BECKMAN CytoFLEX) to calculate the apoptotic cells.

### Reactive oxygen species (ROS) measurement

Intracellular ROS levels were measured using the fluorescent probe DCFH-DA as described previously [23]. Briefly, the stimulated prostate cancer cell lines were exposed to 25 μM DCFH-DA for 30 min in dark. Then, Cells were subjected to flow cytometry (BECKMAN CytoFLEX) to monitor the fluorescence.

### Determination of intracellular labile iron

For the cellular labile iron assay, indicated cells were plated in 60 mm dish at the density of 5 × 10^5^ cells and treated with S.C or DMSO for 48 h. Then, cells were washed with HBSS three times and stained with Mito-FerroGreen (5.0 μM) in HBSS for 30 min at 37 ℃ in the dark. Cells were subjected to flow cytometry (BECKMAN CytoFLEX) to monitor the fluorescence. Intracellular labile iron level was quantified with the MFI value.

### Transmission electron microscopy

PC3 and DU145 cells were treated with S.C or DMSO for 24 h. Cells collected by trypsinization were fixed with 2.5% glutaraldehyde, followed by 1% OsO4. After dehydration, thin sections were stained with uranyl acetate and observed under a transmission electron microscope (JEM-1230, JEOL, Japan).

### Immunohistochemistry and histology

The xenograft tumor samples were collected and fixed in paraformaldehyde overnight. Samples were washed with PBS and dehydrated with ethanol, followed by embedding, sectioning and staining. For Primary antibodies used for immunohistochemical staining were as follows: rabbit polyclonal anti-HMOX1 (1:50; Abclonal) and rabbit monoclonal anti-ki67 (1:100; Abclonal, Lebanon, NH). Sections were also stained with hematoxylin–eosin. Images were obtained at 200 ×magnification on an Olympus microscope.

### RNA-sequencing (RNA-Seq) and bioinformatics analysis

DU145 or PC3 cells were seeded into 100 mm dishes and were treated with S.C (1 μM) or DMSO for 12 h. Then, total RNA was extracted by trizol and was used for RNA-Sequencing by Genewiz-Azenta (Suzhou, China). 3 biological replicates were prepared for each sample. The differentially expressed genes were defined with a cutoff of fold change (FC) of ≥ 2 and the q value < 0.05.

### Xenograft experiments/subcutaneous tumor model

All animal studies and procedures have been approved and performed in accordance with the Animal Care Welfare Committee of Lanzhou University Second Hospital Ethics approval and consent to participate (D2023-136). The CRPC cell line DU145 was used to build the subcutaneous tumor model. DU145 cells were harvested and suspended in PBS at a density of 2 × 10^7^ cells/mL and mixed with matrix gel at a ratio of 1:1. Then, 50 μl suspension was implanted subcutaneously into the flank of 6–8 weeks old NCG (NOD-Prkdc^em26^Il2rg^em26^/Gpt) (GemPharmatech^™^) male mice for 2 weeks. Once these mice developed palpable tumors, mice were randomly divided into three groups: the control group, S.C (2.5 mg/kg) and S.C (5.0 mg/kg) treatment group. Mice weight and tumor size were measured every 2 days. After 7 times drug administration (ip, every two days), mice were sacrificed and tumors were dissected for further analysis, including HE staining, protein immunoblotting etc. Tumor volumes were measured by calipers and calculated as (length × width^2^)/2.

### Statistical analysis

All statistical analyses were performed using GraphPad Prism 8.0(GraphPad Software, Inc., SanDiego, CA, USA). Data are presented as mean ± SD. Significant differences were examined using student’s t test or one-way ANOVA. Differences were considered statistically significant only when p < 0.05.

## Results

### Effect of compounds on DU145 and enzalutamide resistance prostate cancer cells viability

Initially, we constructed a small molecular library targeting DNA damage/repair, angiogenesis, chromatin/epigenetic regulation, cytoskeletal signaling, JAK/STAT signaling, and other pathways, based on FDA and CFDA approved small molecular drugs. To evaluate the cytotoxic effects of these compounds (D1-D98) on prostate cancer cell lines, we exposed DU145 cells to 10 μM compounds for 48 h and assessed cell survival rates using the CCK8 assay. Our results indicated that a few compounds targeting these pathways significantly reduced the viability and survival rates of DU145 cells (Additional file 1: Fig. S1). Notably, compounds targeting histone deacetylase, SIRT1, JNK, and CDKs markedly weakened in DU145 viability. It was consistent with previous studies demonstrating that these molecular play a vital role in cancer initiation, progression and drug resistance [24–28]. Additionally, we established enzalutamide-resistant prostate cancer cells, LNCaP-enz and 22RV1-enz, and evaluated the cytotoxicity of these compounds on these cell lines. Following 48 h of treatment with 5 μM compounds, cells survival and viability were assessed with CCK8 assay. Consequently, we observed that fewer than ten compounds demonstrated cytotoxic effects on DU145, LNCaP-enz, and 22RV1-enz cells (Additional file 1: Fig. S2).

### Effect of compounds and docetaxel on DU145 cells viability

Docetaxel is frequently employed in the treatment of advanced prostate cancer. To explore whether targeting specific pathways could augment the cytotoxic effect of docetaxel on prostate cancer cells, we exposed DU145 cells to 5 μM compounds along with 2.5 nM docetaxel. The findings revealed that compounds D76, D44, D45, and D55 enhanced the cytotoxic impact of docetaxel on DU145 cells. Moreover, these compounds exhibited cytotoxic effects on LNCaP-enz, 22RV1-enz, and DU145 cells.

### S.C suppress prostate cancer cell viability, colony formation and migration abilities

**Fig. 1:**
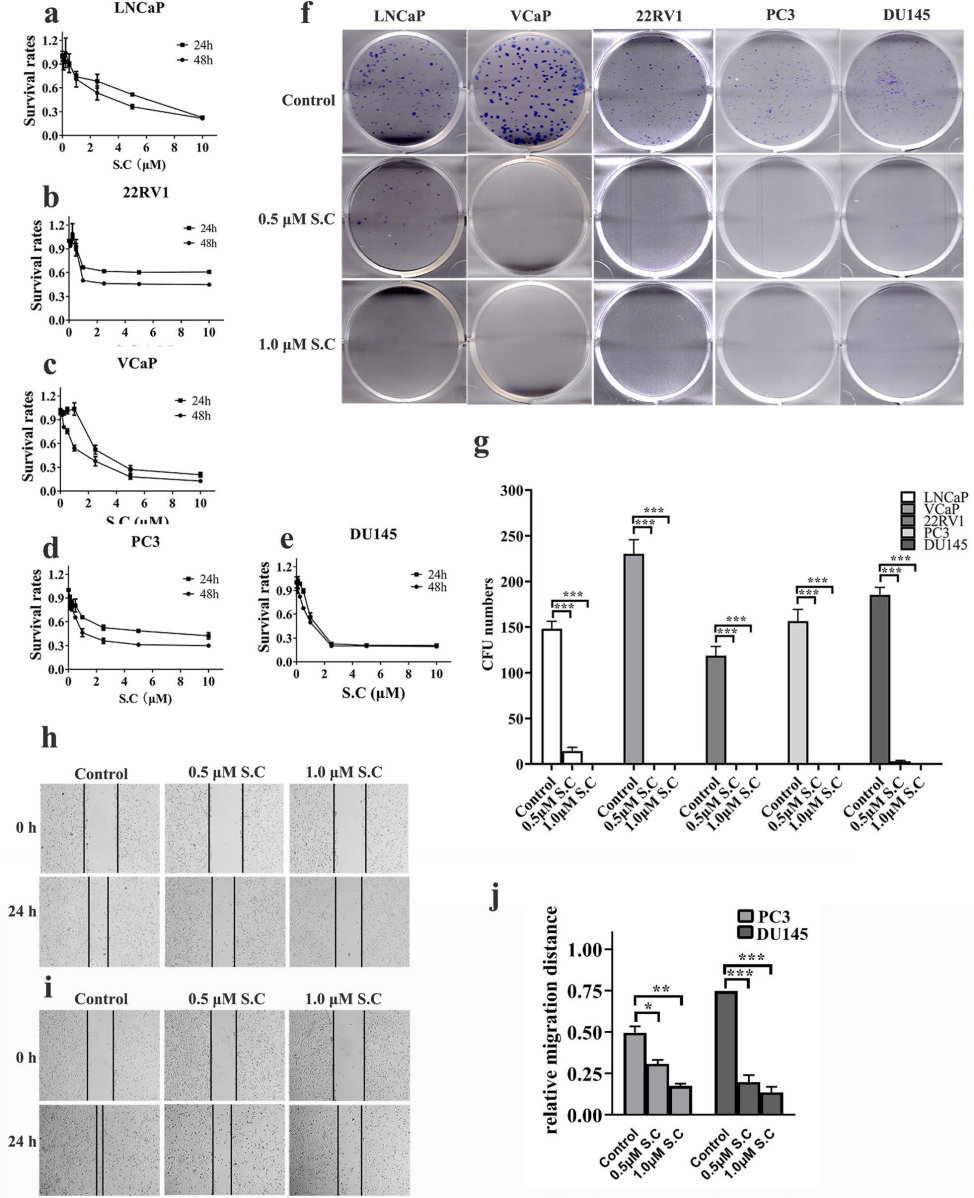
S.C suppresses prostate cancer cell viability, colony formation and migration abilities. **a**–**e** Cell viability of LNCaP, 22RV1, VCaP, PC3 and DU145 cells were measured by CCK8 assay after treatment with indicated concentration of S.C for 24 h and 48 h. **f** Representative images of cell colonies after 10 days treatment with S.C (0, 0.5, 1.0 μM). **g** The number of colonies of S.C-treated LNCaP, 22RV1, VCaP, PC3 and DU145 cells. **h**–**i** Wound healing assay was performed in PC3 and DU145 cells treated with S.C(0, 0.5, 1.0 μM) at 0 h and 24 h. **j** The relative migration distance of S.C-treated PC3 and DU145 cells

S.C is a natural compound that has been supplemented in a number of gingival health products to suppress the growth of microbe. D76 (Sanguinarine chloride, S.C) exhibited cytotoxicity towards DU145, LNCaP-enz, and 22RV1-enz cells and augment the cytotoxic effect of docetaxel. However, its impact on prostate cancer cell growth and the underlying molecular mechanisms remain unclear. To comprehensively assess the cytotoxic effects of S.C on prostate cancer cell viability, we treated LNCaP, 22RV1, VCaP, PC3, and DU145 cells with varying concentrations of S.C for 48 h and evaluated cell viability using the CCK8 assay. The results showed a concentration-dependent reduction in cell viability upon S.C treatment (Fig. 1a–e).Furthermore, we examined its influence on colony formation in these cell lines (LNCaP, 22RV1, VCaP, PC3, and DU145). As depicted in Fig. 1f–g, S.C significantly inhibited clonogenic growth across different prostate cancer cell types. To investigate its impact on cell migration, a wound healing assay was conducted. The findings confirmed that S.C notably suppressed the migration of DU145 and PC3 cells in a dose-dependent manner (Fig. 1h–j).These results suggest that S.C exhibits anti-tumor properties not only in hormone-sensitive prostate cancer cells but also in castration-resistant prostate cancer cells.

### S.C triggers intrinsic apoptosis in prostate cancer cells

**Fig. 2:**
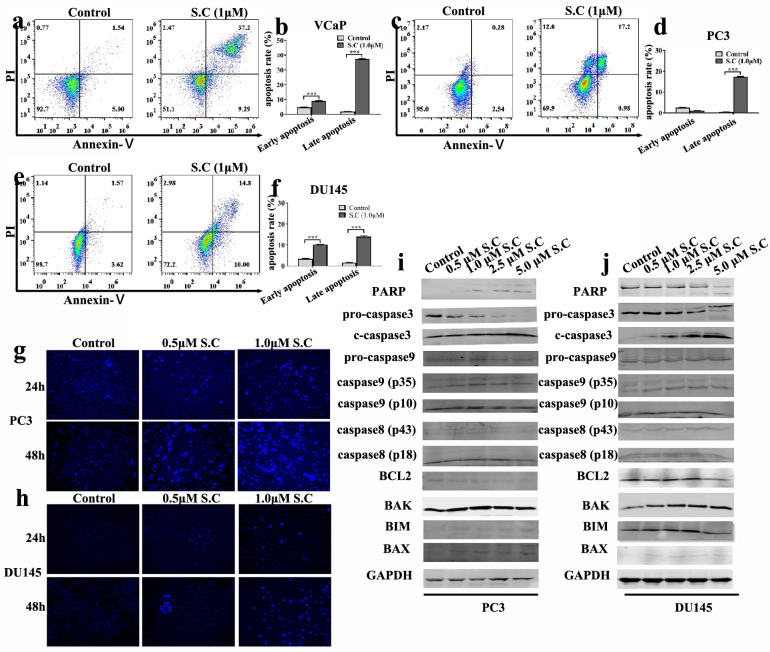
S.C triggers intrinsic apoptosis in prostate cancer cell. After the cells were treated with 1.0 μM S.C for 48 h, apoptosis rate of VCaP **a**, **b**, PC3 **c**, **d** and DU145 **e**, **f** cells was detected by flow cytometry. After the cells were treated with 0, 0.5,1.0 μM S.C for 24 h or 48 h, Hoechst 33,258 staining was used to observe the morphological changes of PC3 **g** and DU145 **h** cells. Intrinsic and extrinsic apoptosis related proteins level in PC3 **i** and DU145 **j** cells receiving 0, 0.5,1.0 μM S.C treatments for 48 h

Previous studies have reported sanguinarine's ability to induce apoptosis. Therefore, we conducted an assessment of apoptosis in prostate cancer cells treated with S.C for 48 h compared to untreated cells. Annexin V/propidium iodide (PI) staining revealed a significant increase in apoptosis rates in S.C-treated VCaP, PC3, and DU145 cells. Particularly, a substantial proportion of late-stage apoptotic cells was observed within the apoptotic cell population (Fig. 2a–f).Moreover, PC3 and DU145 cells treated with S.C were stained with Hoechst 33,258 dye at 24 and 48 h to observe nuclear condensation. Result showed S.C treatment increased nuclear condensation in both PC3 and DU145 cells (Fig. 2g–h).Furthermore, immune blot analysis demonstrated that S.C stimulation led to increased cleavage of PARP (caspase3 substrate), caspase3, and caspase9, while no significant effect on caspase8 was observed. Additionally, BCL2 levels exhibited a slight reduction, whereas BAK and BAX levels were slightly increased in the S.C-treated cells. These findings collectively suggest that S.C triggers intrinsic apoptosis in prostate cancer cells.

### S.C-induced cell death partly depends on ferroptosis

**Fig. 3:**
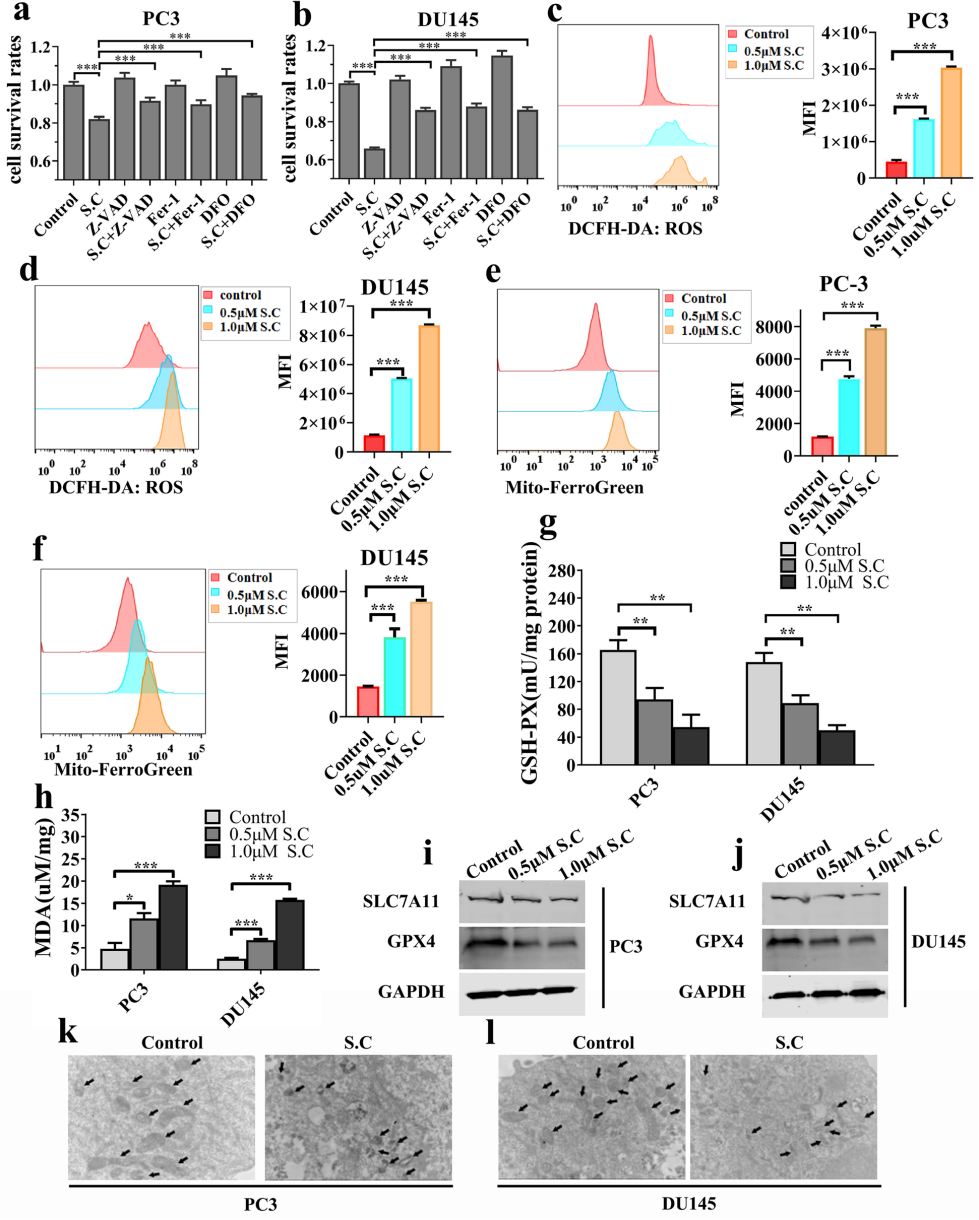
S.C-induced cell death depends on ferroptosis. PC3 **a** and DU145 **b** cells were treated with S.C (0.5 μM) with or without Z-VAD-FMK (20 μM), fer-1 (2.0 μM), and DFO (5.0 μM) for 48 h. Then, Cell survival rates were measured using CCK8 assay. PC3 **c**, **e** and DU145 **d**, **f** cells were treated with S.C (0.5, 1.0 μM) for 48 h, intracellular ROS, ferrous ion (Fe^2+^), MDA **g** and GSH-PX **h** level were detected. **i**, **j** Western blotting analysis of SLC7A11 and GPX4 levels in PC3 and DU145 cells receiving S.C treatment or not for 48 h. **k**, **l** TEM images of the mitochondrial structure in PC3 and DU145 cells treated with or without S.C (0.5 μM)for 24 h

A recent study revealed that S.C induces ferroptosis. Further analysis through FCAS indicated that apoptosis might not be the sole mechanism behind S.C-induced cell death. To elucidate the specific mechanism of cell death, we inhibited apoptosis using z-VAD-fmk and observed that z-VAD-fmk treatment only partially rescued S.C-induced cell death (Fig. 3a–b).Blocking ferroptosis with fer-1 or DFO in S.C-treated cells resulted in increased cell survival rates, indicating the involvement of ferroptosis in S.C-induced cell death. Ferroptosis is an iron-dependent oxidative cell death process initiated by ROS from the Fenton reaction and subsequent lipid peroxidation [29]. Therefore, we evaluated ROS levels and mitochondrial labile free iron (Fe2 +) upon S.C treatment, which unsurprisingly showed an increase (Fig. 3c–f).Furthermore, S.C treatment resulted in a significant rise in MDA levels and a decrease in GSH-PX levels in prostate cancer cells (Fig. 3g–h). Protein levels of SLC7A11 and GPX4 were notably reduced in the S.C treatment condition (Fig. 3i–j). Consistent with these findings, transmission electron microscopy (TEM) analysis displayed distinct dense and shrunken mitochondria in S.C-treated PC3 and DU145 cells (Fig. 3k–l). Collectively, these data strongly suggest that ferroptosis is a contributing factor to S.C-induced cell death.

### S.C targets regulation of HMOX1 expression

**Fig. 4:**
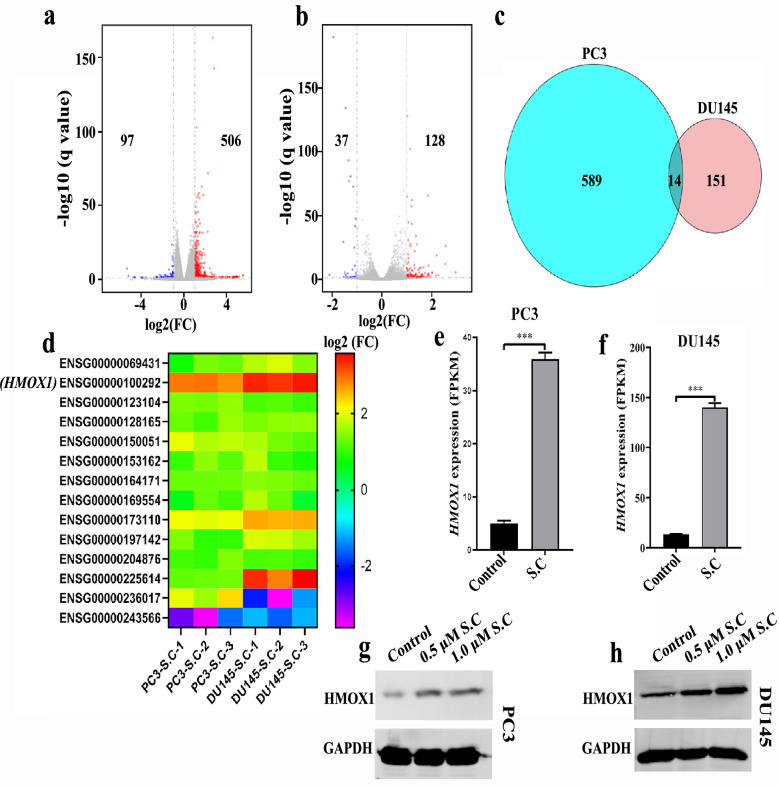
S.C targets regulation of HMOX1 expression Volcano plots for differentially expressed genes in PC3 **a** and DU145 **b** cells receiving 1.0 μM S. **C** treatment for 12 h or not. c Intersections of differentially expressed genes with in PC3 and DU145 cells. **d** List of differentially expressed genes in DU145 and PC3 cells. mRNA expression level of HMOX1 in PC3 and DU145 cells receiving S.C treatment or not (**e**, **f**). Protein level of HMOX1 in PC3 and DU145 cells receiving S.C treatment or not (**g**, **h**)

To unravel the mechanism underlying S.C-triggered ferroptosis, we exposed PC3 and DU145 cells to S.C for 12 h for RNA sequencing analysis. The results revealed 603 differentially expressed genes in PC3 cells and 165 in DU145 cells (Fig. 4a–b). Notably, fourteen genes exhibited differential expression in both PC3 and DU145 cells (Fig. 4c). Among these, the expression of HMOX1 (ENSG00000100292) showed the most prominent increase (Fig. 4d). HMOX1 is known to drive ferroptosis by metabolizing heme into iron, thereby promoting Fe2^+^ overload. Further analysis of the RNA-seq data highlighted significantly elevated HMOX1 mRNA levels in S.C-treated PC3 and DU145 cells (Fig. 4e–f). Western blotting also corroborated that S.C treatment increased HMOX1 protein levels (Fig. 4g–h). These collective findings strongly suggest that S.C-induced ferroptosis may rely on Fe2^+^ overload facilitated by HMOX1 protein.

### S.C-induced HMOX1 up-regulation contributes to ferroptosis in prostate cancer cells

**Fig. 5:**
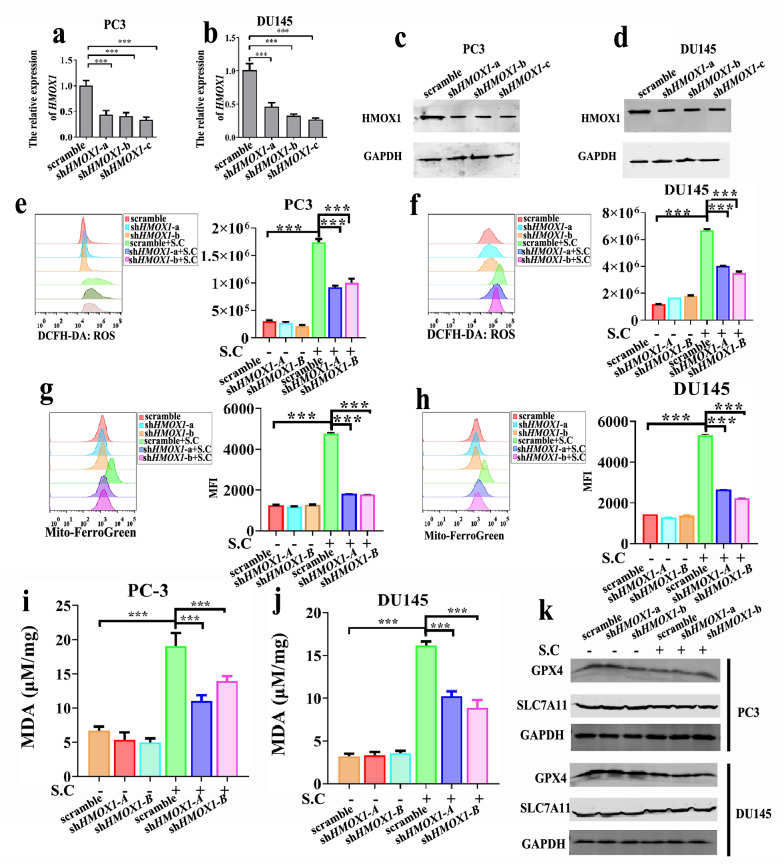
S.C inducing HMOX-1 up-regulation contributes to ferroptosis in prostate cancer cell. mRNA and protein levels of HMOX1 measured by qRT-PCR and western blot in PC3 *HMOX1* knockdown **a**, **c** and DU145 *HMOX1* knockdown cells **b**, **d**. DCFH-DA probe was used to detected ROS levels in PC3 *HMOX1* knockdown **e** and DU145 *HMOX1* knockdown **f** cells with S.C treatment or not. Mito-FerroGreen probe was used to detected iron levels in PC3 *HMOX1* knockdown **g** and DU145 *HMOX1* knockdown (h) cells. **i**, **j** Quantification of the MDA levels in *HMOX1* knockdown cells. GPX4 and SLC7A11 protein level in PC3 *HMOX1* knockdown **k** and DU145 *HMOX1* knockdown **l** cells

To delineate the role of HMOX1 in S.C-induced ferroptosis, *HMOX1*knockdown was performed in PC3 and DU145 cells.As depicted in Fig. 5a–d, the levels of HMOX1 mRNA and protein markedly decreased in shHMOX1-a/b/c cells. Notably, the reduction of *HMOX1* also led to a significant decrease in S.C-induced ROS production in PC3 and DU145 cells (Fig. 5e–f). Additionally, *HMOX1* knockdown partially attenuated S.C-induced MDA generation (Fig. 5 g–h) and mitigated cellular Fe^2+^ levels (Fig. 5i). These observations suggest that *HMOX1* knockdown impedes S.C-induced ferroptosis in prostate cancer by mitigating Fe^2+^ overload, diminishing ROS levels, and alleviating lipid peroxidation. It was established that S.C could downregulate the expression of GPX4 and SLC7A11 (Fig. 3i–j). We sought to investigate whether GPX4 and SLC7A11 levels were influenced by HMOX1. However, the Western blotting results revealed that the knockdown of *HMOX1* did not rescue the levels of GPX4 and SLC7A11 in S.C-treated cells, indicating that the suppression of GPX4 and SLC7A11 expression by S.C was independent of HMOX1. These findings suggest that the increased expression of HMOX1 induced by S.C contributes to ferroptosis in prostate cancer cells.

### S.C-induced HMOX1 up-regulation depends on decreasing the stability of BACH1 protein

**Fig. 6:**
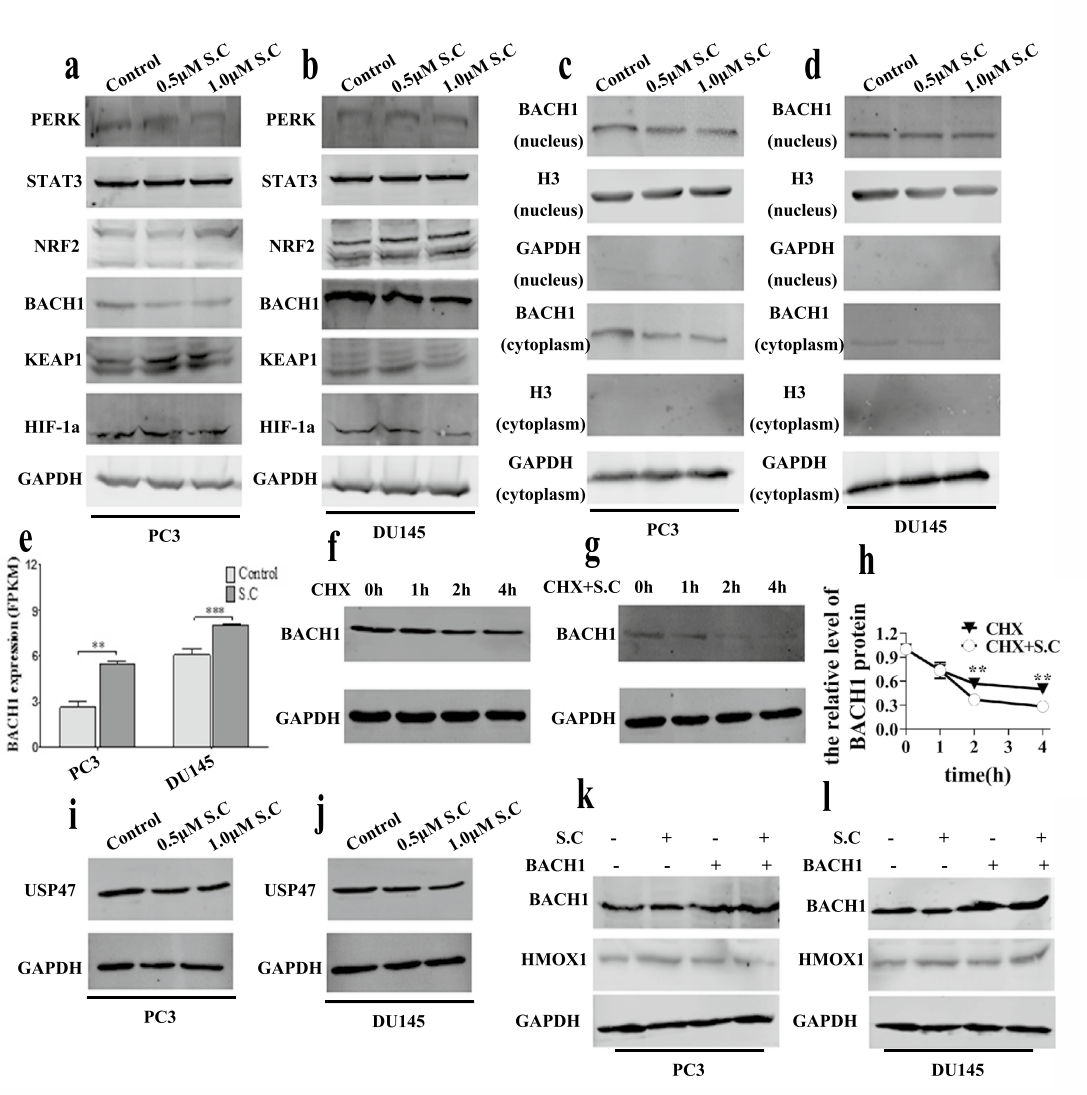
S.C induces HMOX1 up-regulation depending on decreasing the stability of BACH1 protein. Western blotting analysis of PERK, STAT3, NRF2, BACH1, KEAP1, and HIF-1α levels in PC3 **a** and DU145 **b** cells receiving S.C (0, 0.5, 1.0 μM) treatment for 48 h. Immunoblotting analysis of BACH1 in nuclear and cytoplasm of S.C (0, 0.5, 1.0 μM)-treated PC3 **c** and DU145 **d** cells. **e** mRNA expression level of BACH1 in S.C-treated PC3 and DU145 cells. **f**–**g** Cycloheximide (CHX) chase assay in S.C-treated DU145 cells. **h** BACH1 protein levels were calculated by quantification of BACH1protein levels. Immunoblotting analysis of USP47 in S.C (0, 0.5, 1.0 μM)-treated PC3 **i** and DU145 **j** cells. Immunoblotting analysis of BACH1 and HMOX1 in S.C (0, 1.0 μM)-treated PC3 **k** and DU145 **l** cells transiently expressing empty vector or BACH1 vector

Various cellular stress conditions can trigger *HMOX1* transcription due to the presence of a stress-responsive element (StRE) in the *HMOX1* gene enhancer. Under normal conditions, BACH1 represses *HMOX1* transcription by binding to the *HMOX1* enhancer with small MAF proteins. Stimulation with heme or arsenite induces *HMOX1* transcription by inactivating BACH1 and promoting NRF2 interaction with the *HMOX1* promoter [30, 31]. Additionally, PERK, STAT3 and HIF-1a have been found to promote the expression of HMOX1 [32–35]. However, our results indicated that S.C-induced HMOX1 expression might not rely on PERK, STAT3, or HIF-1α (Fig. 6a–b). Instead, down-regulation of BACH1 appeared to play a significant role in S.C-induced HMOX1 expression (Fig. 6a–b).To unravel the underlying mechanism, we analyzed BACH1 levels in the nuclear and cytoplasm. Our findings revealed that S.C treatment significantly decreased nuclear BACH1 levels and slightly reduced cytoplasmic BACH1 levels (Fig. 6c–d).Further analysis of the RNA-seq data unexpectedly showed an increase in BACH1 mRNA expression upon S.C treatment (Fig. 6e). This discrepancy suggested that S.C represses BACH1 protein expression by decreasing BACH1 protein stability. To validate this hypothesis, we performed a Cycloheximide (CHX) chase experiment in DU145 cells in the presence or absence of S.C. The results demonstrated that S.C treatment notably reduced BACH1 protein stability (Fig. 6f–h). Previous research has highlighted the role of ubiquitin carboxyl-terminal hydrolase 47 (USP47) in stabilizing BACH1 through deubiquitylation [36]. Hence, we explored whether USP47 played a role in S.C-repressed BACH1 protein expression. Intriguingly,we found that S.C treatment reduced USP47 levels in cells (Fig. 6i–j). These findings suggest that S.C might decrease BACH1 protein stability and subsequently promote HMOX1 expression in a USP47-dependent manner. Subsequently, we treated cells over-expressing BACH1 with S.C and observed that BACH1 over-expression could reduce HMOX1 expression under S.C-treated conditions (Fig. 6k–l). This suggests that modulating BACH1 levels can influence HMOX1 expression, providing further insights into the regulatory mechanism of S.C-induced HMOX1 expression. These collective findings strongly indicate that the upregulation of HMOX1 induced by S.C is intricately linked to the reduction in BACH1 protein stability, potentially facilitated through USP47-mediated deubiquitylation.

### ROS is responsible for BACH1 down-regulation in S.C inducing ferroptosis

**Fig. 7:**
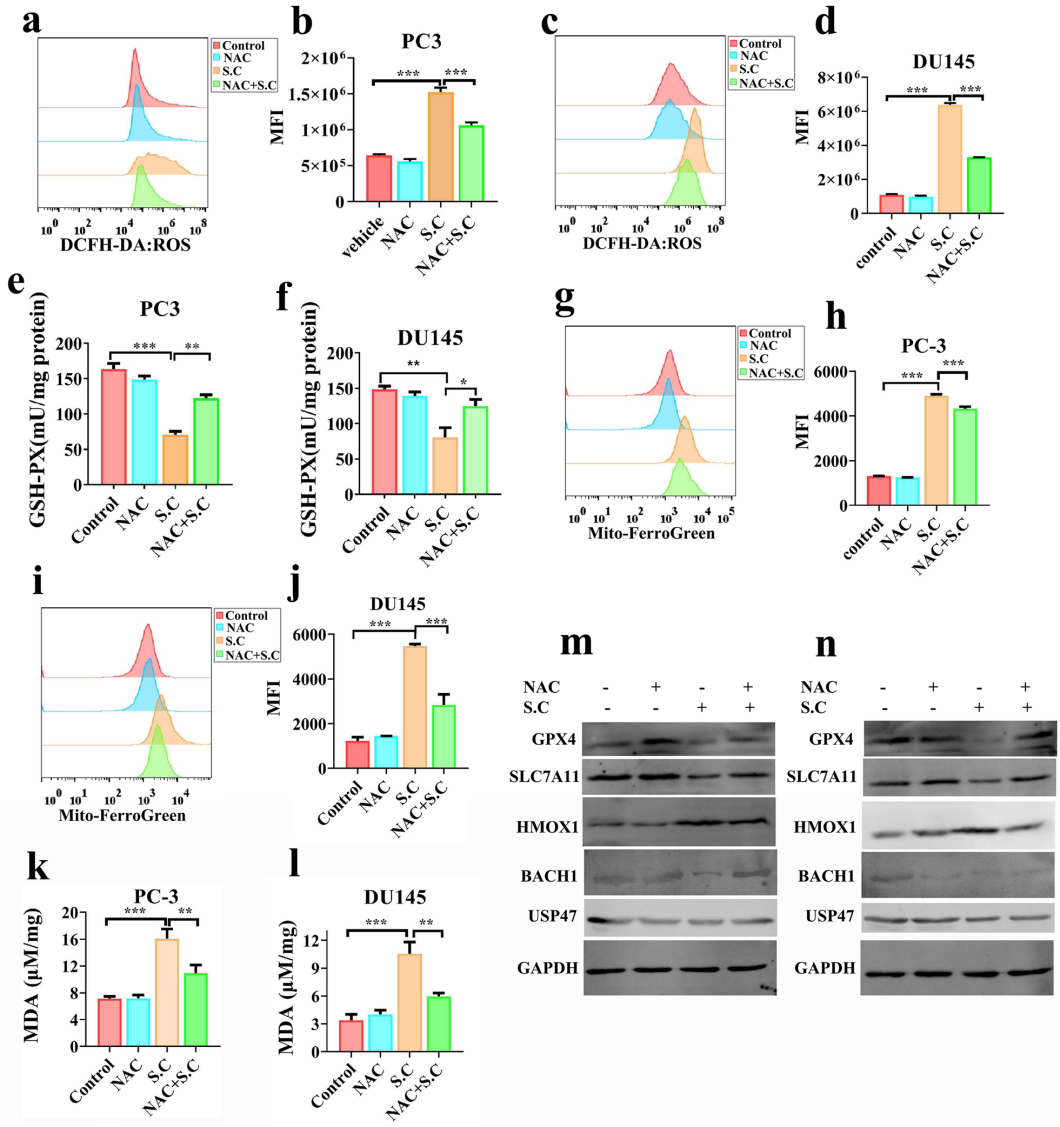
ROS is responsible for BACH1 decreasing in S.C inducing ferroptosis. Cells were pretreated with vehicle or 5 mM NAC for 1 h before treatment with 0.5 μM S.C in PC3 **a**, **b**, **g**, **h** or DU145 **c**, **d**, **i**, **j** cells. After 24 h treatment with vehicle or S.C in the presence of NAC, cells were stained with DCFH-DA or Mito-FerroGreen for flow cytometry assay. After 48 h treatment with vehicle or S.C in the presence of NAC, cells were used to analyze the contents of GSH, MDA in PC3 **e**, **k** and DU145 cells **f**, **l**. After 48 h treatment, GPX4, SLC7A11, HMOX1, BACH1 and USP47 levels were analyzed by Immunoblotting in PC3 **m** and DU145 cells **n**

ROS generation has been established to mediate sanguinarine-induced apoptosis and ferroptosis [13]. HMOX1 is notably expressed under oxidative stress conditions [31]. To investigate the potential contribution of ROS generation to HMOX1 upregulation in S.C-induced ferroptosis, we co-treated cells with the ROS scavenger NAC (N-acetyl-L-cysteine) alongside S.C. The results demonstrated that NAC significantly reduced S.C-induced ROS generation in PC3 and DU145 cells (Fig. 7a–d). Furthermore, NAC treatment partially increased GSH-PX levels in the cells (Fig. 7e–f). When compared to cells treated solely with S.C, the levels of labile free iron and MDA significantly decreased in prostate cancer cells co-treated with S.C and NAC (Fig. 7g–l). Immunoblotting analysis confirmed that inhibiting ROS generation with NAC partially rescued the S.C-mediated expression alterations of GPX4, SLC7A11, BACH1, and USP47. Additionally, NAC treatment attenuated S.C-induced HMOX1 expression in prostate cancer cells (Fig. 7m–n). These findings collectively suggest that S.C may activate the ROS/USP47/BACH1/HMOX1 signaling pathway, ultimately leading to ferroptosis in prostate cancer cells.

### S.C-induced ferroptosis impedes prostate tumor growth in vivo

**Fig. 8:**
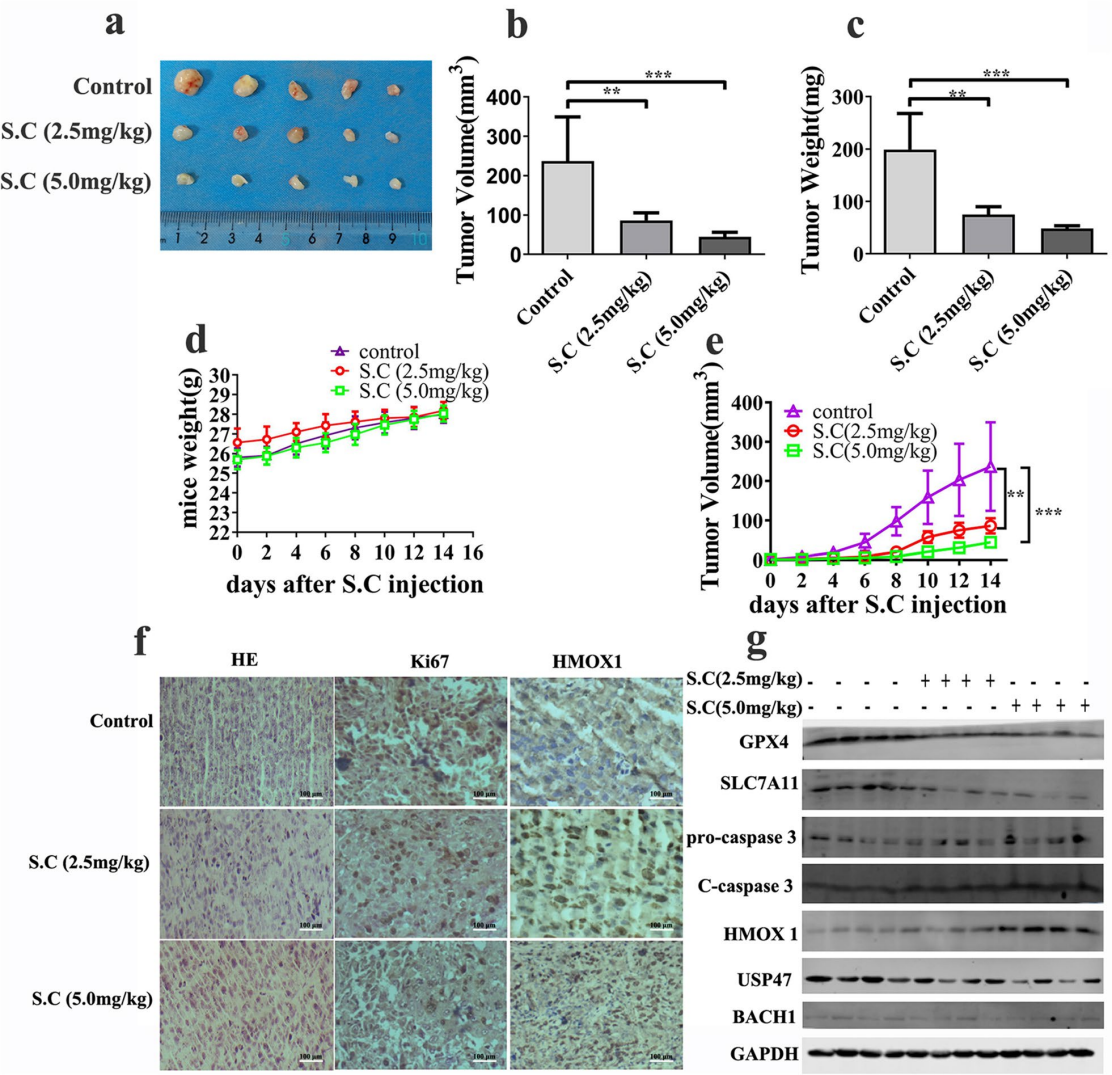
S.C treatment inhibits cancer progression in DU145 xenograft model. DU145 cells were subcutaneously injected into the flank of NCG nude mice. 10 days after inoculation, the mice were treated with S.C (2.5, 5.0 mg/kg body weight) via the intraperitoneal (i.p) route every 2 days. **a** 2 weeks after injection, tumor tissues were excised and photographed. The excised tumor weight **b** and volume **c** were calculated. **d** Body weight was measured every 2 days after administration of S.C. **e** Tumor volume was calculated every 2 days after administration of S.C. **f** Representative images of H&E and immunohistochemical staining for Ki67 and HMOX1 **g** protein levels of GPX4, SLC7A11, caspase3, HMOX1, USP47 and BACH1 measured by western blot

To further understand the impact of S.C on prostate tumor growth in vivo, we established a xenograft animal model by subcutaneously injecting DU145 cells into NCG (NOD/ShiLtJGpt-*Prkdc*^*em26Cd52*^*Il2rg*^*em26Cd22*^/Gpt) mice. Once the mice developed palpable tumors, they were intraperitoneally administered with S.C (2.5 mg/kg or 5.0 mg/kg) or an equivalent volume of saline every two days for a total of seven times drug administrations. As depicted in Fig. 8a, the S.C treatment notably suppressed tumor growth. Both the size and weight of the tumors were significantly lower compared to the control group (Fig. 8a–c, e).Immunohistochemistry analysis revealed a significant reduction in the expression of the proliferation marker protein Ki67 in the S.C treatment group compared to the control group. Additionally, HMOX1 expression was upregulated in the S.C treatment group (Fig. 8f). Proteins extracted from these subcutaneously transplanted tumors were subjected to immunoblotting, confirming that S.C treatment initiated ferroptosis in prostate tumors. The S.C treatment group exhibited relatively lower levels of GPX4 and SLC7A11 compared to the control group. Consistent with the in vitro findings, HMOX1 expression was noticeably increased in the S.C treatment group (Fig. 8g).Overall, these results strongly indicate that S.C-induced ferroptosis attenuates prostate tumor growth both in vitro and in vivo.

## Discussion

Different candidate treatment options are available for prostate cancer patients, depending on the disease stage. For localized/locally advanced PCa, radical prostatectomy and radiotherapy are the standard treatment approaches. For metastatic androgen-sensitive PCa, androgen deprivation therapy (ADT) is standard treatment. However, drug resistance is inevitable and most patients develop into lethal metastatic castration-resistant prostate cancer (mCRPC). Until now, CRPC remains incurable [37]. The inactivation of apoptosis is central to the development of CRPC. Several anti-apoptotic members of the *bcl-2* gene family, including BCL2, BCL-X, and MCL-1 showed high expression during the progression of prostate cancers [38, 39]. ADT treatment also up-regulate the expression of anti-apoptotic proteins. For instance, androgens repress BCL2 expression via negatively modulating the activities of the E2F site in its promoter through activating the CDKI-RB axis [40]. ADT treatment significantly increases the expression of oncogene *bcl-2*. The high expression of these anti-apoptotic proteins could effectively prevent ADT-induced apoptosis in prostate cancer [39]. Distinct from apoptosis, necroptosis, and pyroptosis, ferroptosis is a type of regulated cell death (RCD) characterized by intracellular iron accumulation and lipid peroxidation [7]. Extensive studies suggest that ferroptosis plays a pivotal role in tumor suppression and reversing drug resistance [9, 41].Targeted ferroptosis with its inducer significantly halted the tumor growth of treatment-resistant prostate cancer. The combination of a ferroptosis inducers (FINs) with enzalutamide or abiraterone acetate is highly synergistic for inducing advanced prostate cancer cell death in vitro and in vivo [42]. Targeted ferroptosis is a promising therapeutic approach to overcome drug resistance in prostate cancer [43]. Regretfully, there is still no specific ferroptosis inducer currently applied in clinical treatment.

In this study, we constructed a small molecular library targeting DNA damage/repair, angiogenesis, chromatin/epigenetic regulation, cytoskeletal signaling, JAK/STAT signaling, and other pathways. Based on screening of these small molecules, we found that S.C inhibited the activity of prostate cancer cells, including enzalutamide-resistant prostate cancer cells. Additionally, it enhances docetaxel’s cytotoxic effect on prostate cancer cells. Sanguinarine is a natural product extracted from the roots of Sanguinaria canadensis. Studies have reported that sanguinarine promoted cellular glutathione depletion [16, 44]. This suggests that sanguinarine may induce cell death through ferroptosis. Recently, two groups reported that sanguinarine facilitates ferroptosis in non-small cell lung cancer and cervical cancer [13, 45]. However, the mechanism by which sanguinarine triggers ferroptosis remains unclear. In vitro experiments also showed that S.C repressed colony formation and migration in prostate cancer cells. S.C treatment activated intrinsic apoptosis pathway in prostate cancer cells. However, pan-caspase inhibitor z-VAD-fmk only partially prevented S.C-induced cell death. Further studies demonstrated that ferrostatin-1 (fer-1) and deferoxamine (DFO) also partially decreased the cytotoxic activity of S.C in prostate cancer cells. S.C reduced the level of GSH and the expression of GPX4 and SLC7A11. MDA and Fe^2+^ levels increased in S.C-treated prostate cancer cells. All results indicated ferroptosis was involved in S.C-induced cell death. Consequently, RNA-seq analysis of S.C treated cell identified HMOX1 as a possible target of S.C. HMOX1 is a stress-induced enzyme that metabolizes heme into carbon monoxide, iron, and biliverdin. Studies showed that HMOX1 mediated ferroptosis by promoting ROS production and iron accumulation [46, 47]. We indeed found that S.C induced a high expression of HMOX1 in mRNA and protein levels, and HMOX1 knockdown decreased ROS, MDA and iron generation in S.C-treated cells. Consistent with previous research, we found the expression of SLC7A11 and GPX4 did not rely on HMOX1 (Fig. 4k–j). This suggests that S.C may facilitate ferroptosis by attenuating GSH generation and enhancing Fe^2+^ overload in prostate cancer cells. Further analysis of transcription factors expression, revealed thatHMOX1 may contribute to S.C-induced ferroptosis. BACH1 down-regulation appears to be involved in S.C-induced ferroptosis as S.C treatment decreased BACH1 expression in nucleus and cytoplasm. BACH1 overexpression abolished S.C-induced HMOX1 expression. Further studies showed that the S.C-reduced BACH1 protein levels relied on decreasing its stability. BACH1 stability was mediated by USP47 via enhancing the deubiquitylation of BACH1 [36]. Herein, we also found that S.C attenuated USP47 expression in prostate cancer cells. This implies that S.C may increase HMOX1 expression by decreasing BACH1 stability in a USP47 dependent manner. Extensive evidence has identified that ROS induced ferroptosis [48–50]. Our study demonstrated that S.C-induced ferroptosis also depended on ROS accumulation. ROS scavenger NAC significantly decreased iron, MDA levels and enhanced BACH1-mediated HMOX1 transcriptional inhibition effects in S.C treated cells. Finally, in vivo experiments showed that S.C delayed tumor progression. Immunohistochemistry analysis confirmed that the tumor suppressive effect of S.C may indeed rely on activating ROS/USP47/BACH1/HMOX1 axis.

## Conclusion

Our work clarified that S.C effectively inhibits the viability of PCa and CRPC cells, overcome enzalutamide resistance, and potentially enhances the cytotoxicity of docetaxel. Furthermore, the results of pharmacological action and mechanism analysis revealed that the anti-tumor effects of S.C are mediated by both apoptosis and ferroptosis. More specifically, our study revealed that S.C represses tumor progression and induces ferroptosis in prostate cancer cells by targeting the ROS/USP47/BACH1/HMOX1 axis. These findings offer valuable insights into the mechanistic understanding of how S.C represses the progression of prostate cancer. Additionally, targeting ferroptosis with S.C may present a novel opportunity for prostate cancer therapy.

### Supplementary Information

**Additional file 1:** **Fig.S1 **Effects of compounds on DU145 cells viability.DU145 cells were treated with 10 μM compounds for 48 h. Then, CCK8 assay performed for cell viability assessment. **Fig.S2 **Effects of compounds on ezalutamide resistance prostate cancer cells viability.22RV1-enz and LNCaP-enz cells were treated with 5 μM compounds for 48 h. Then, CCK8 assay performed for cell viability assessment. **Fig. S3 **Effect of compoundsand docetaxel on DU145 cells viability.DU145 cells were treated with 5 μM compounds and 2.5 nM docetaxel for 48 h. Then, CCK8 assay performed for cell viability assessment. **Fig. S4** S.C triggers ferroptosis in prostate cancer cell. Western blotting analysis of SLC7A11 and GPX4 levels in PC3 **a**, **c** and DU145 **b**, **d** cells receiving S.C (0, 0.5, 1.0μM) treatment for 48 h. protein levels of HMOX1 measured by western blot in DU145 HMOX1 knockdown cells **e**. MDA level were detected in PC3 and DU145 cells treated with S.C or erastin for 48h **f**. SLC7A11 and GPX4 levels were detected in PC3 and DU145 cells treated with S.C or erastin for 48h **g**.

## Data Availability

The data used to support the finding of this study are available on request.

## Abbreviations

BACH1: BTB domain And CNC homolog 1
GSH: Glutathione
HMOX1: Heme oxygenase-1
MDA: Malondialdehyde
S.C: Sanguinarine chloride
ROS: Reactive oxygen species
USP47: Ubiquitin-specific peptidase 47
