# Supplementary figures and images for: Sanguinarine chloride induces ferroptosis by regulating ROS/BACH1/HMOX1 signaling pathway in prostate cancer
Source: Chin Med. 2024 Jan 9;19:7. doi: 10.1186/s13020-024-00881-6 (PMC10777654; doi:10.1186/s13020-024-00881-6)

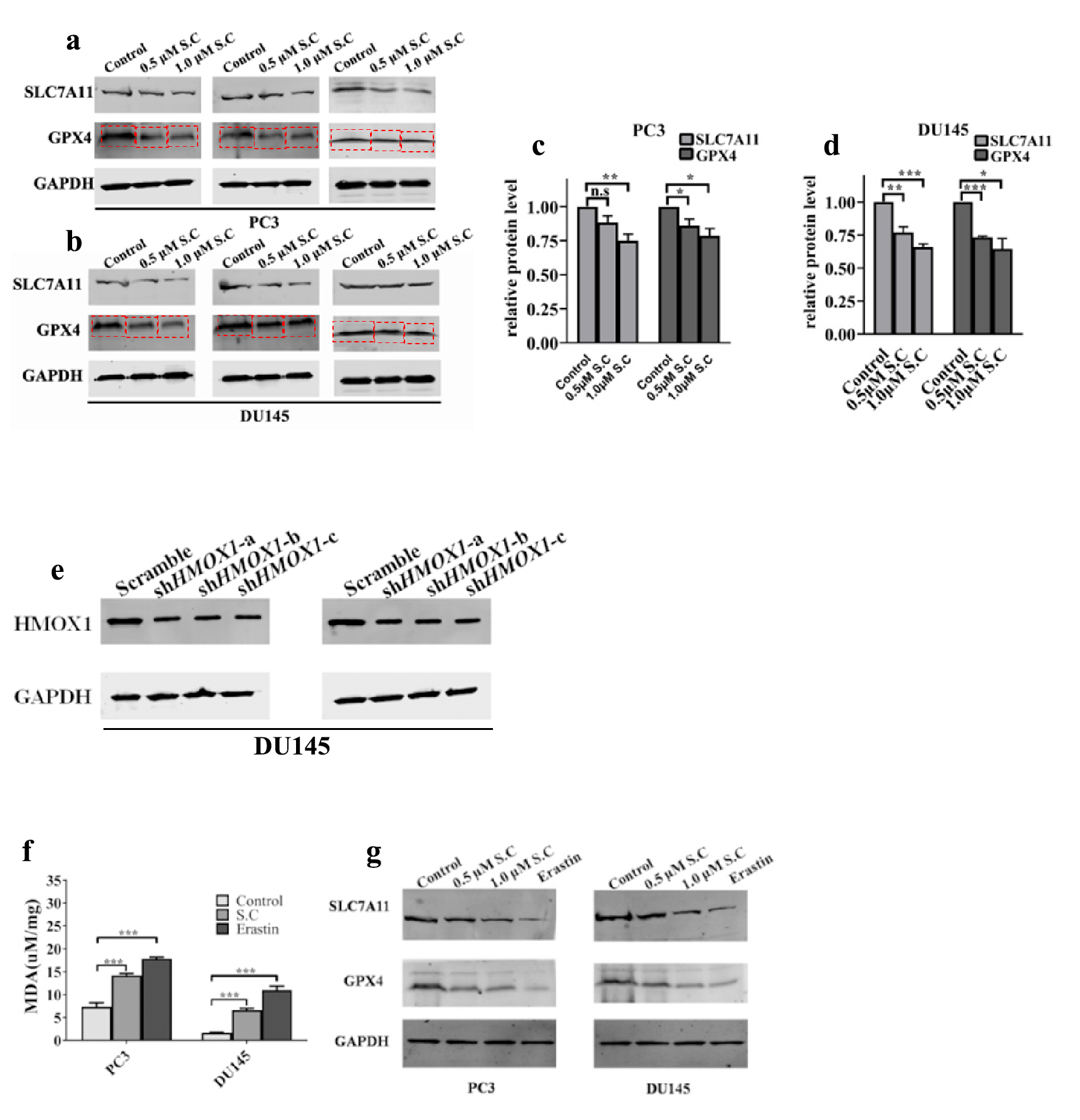

Supplement: Supplementary file 1 — Additional file 1: Fig.S1 Effects of compounds on DU145 cells viability.DU145 cells were treated with 10 μM compounds for 48 h. Then, CCK8 assay performed for cell viability assessment. Fig.S2 Effects of compounds on ezalutamide resistance prostate cancer cells viability.22RV1-enz and LNCaP-enz cells were treated with 5 μM compounds for 48 h. Then, CCK8 assay performed for cell viability assessment. Fig. S3 Effect of compoundsand docetaxel on DU145 cells viability.DU145 cells were treated with 5 μM compounds and 2.5 nM docetaxel for 48 h. Then, CCK8 assay performed for cell viability assessment. Fig. S4 S.C triggers ferroptosis in prostate cancer cell. Western blotting analysis of SLC7A11 and GPX4 levels in PC3 a, c and DU145 b, d cells receiving S.C (0, 0.5, 1.0μM) treatment for 48 h. protein levels of HMOX1 measured by western blot in DU145 HMOX1 knockdown cells e. MDA level were detected in PC3 and DU145 cells treated with S.C or erastin for 48h f. SLC7A11 and GPX4 levels were detected in PC3 and DU145 cells treated with S.C or erastin for 48h g. [file 13020_2024_881_MOESM1_ESM.zip › Additional file/figure S4.tif]
